# Early and late rapid torque characteristics and select physiological correlates in middle-aged and older males

**DOI:** 10.1371/journal.pone.0231907

**Published:** 2020-04-23

**Authors:** Alex A. Olmos, Matthew T. Stratton, Phuong L. Ha, Benjamin E. Dalton, Trisha A. VanDusseldorp, Gerald T. Mangine, Yuri Feito, Micah J. Poisal, Joshua A. Jones, Tyler M. Smith, Garrett M. Hester

**Affiliations:** 1 Department of Exercise Science and Sport Management, Kennesaw State University, Kennesaw, Georgia, United States of America; 2 Department of Kinesiology and Sport Management, Texas Tech University, Lubbock, Texas, United States of America; University of Bourgogne France Comté, FRANCE

## Abstract

**Purpose:**

The purpose of this study was to compare early and late rapid torque parameters of the plantar flexors (PFs) in middle-aged (MM) and older (OM) males, and determine the effect of normalization to peak torque (PT) and muscle cross-sectional area (CSA).

**Methods:**

Twenty-nine healthy, MM (n = 14; 45 ± 2 yrs) and OM (n = 15; 65 ± 3 yrs) performed rapid, maximal isometric contractions of the PFs. PT, as well as rate of torque development and impulse during the early (0–50 ms; RTD_0-50_, IMP_0-50_) and late (100–200 ms; RTD_100-200_, IMP_100-200_) contraction phases were calculated. Torque at 50 (TQ_50_), 100 (TQ_100_), and 200 (TQ_200_) ms was also obtained. CSA and echo-intensity (EI) of the gastrocnemii were acquired via ultrasonography. Torque variables were normalized to PT and CSA. Rate of EMG rise (RER) for the medial gastrocnemius was calculated at 30, 50 and 75 ms.

**Results:**

TQ_100_ (MM = 69.71 ± 16.85 vs. OM = 55.99 ± 18.54 Nm; p = 0.046), TQ_200_ (MM = 114.76 ± 26.79 vs. OM = 91.56 ± 28.10 Nm; p = 0.031), and IMP_100-200_ (MM = 4.79 ± 1.11 vs. OM = 3.83 ± 1.17 Nm·s; p = 0.032) were lower in OM. PT, TQ_50_, RTD_0-50_, IMP_0-50_, RTD_100-200_, RER, CSA, and EI were similar between groups (p > 0.05). No differences were found for normalized torque variables (p > 0.05). EI was moderately associated with normalized torque parameters only (*r* = -0.38 –-0.45). RER, at 75 ms, was moderately correlated with early, absolute torque measures and rapid torque variables made relative to PT and CSA (*r* = 0.41 –-0.64).

**Conclusion:**

Late rapid torque parameters of the PFs were preferentially impaired in OM compared to MM, and PT as well as CSA appeared to mediate this result.

## Introduction

Muscle strength is an important marker of physical health that expresses a moderate decrease from young to middle-age followed by a sharper decline with advanced age (8^th^ decade) [1, 2]. However, increasingly more research has focused on rapid torque (or force) parameters (e.g., rate of torque development (RTD; Δtorque/Δtime)) as markers of neuromuscular function, which emphasize torque production during the initial 200 ms of muscle contraction [3], whereas maximal torque requires at least ~300 ms to be achieved [4]. Rapid torque measures are more dramatically affected by age compared to maximal strength [5–7], although most reports are limited to comparisons between young and older adults. Recent reports demonstrate that RTD is an independent predictor of physical function tasks such chair rise ability, timed “up and go”, as well as casual and maximal walking velocity in older adults [8, 9]. In addition, an age-related decrease in rapid torque production of the support leg within 100–200 ms after tripping is believed to influence the diminished ability to recover from falls in older adults [10]. Impulse, area under the torque-time curve, is another less frequently examined measure of rapid torque production that is more reflective of the torque-time curve history compared to RTD since the latter is derived from a line of best fit. Thus, assessing both RTD and impulse may provide unique insight on rapid torque capacity, however, reports on age-related changes in impulse between middle-aged and older adults are limited [11].

Typically, rapid torque measures are examined at different time intervals of a muscle contraction such as the early (i.e., 0–50 ms) and late phase (i.e., 100–200 ms) [6, 12]. Early rapid torque variables are associated with initial motor unit recruitment and firing rates [5, 13], as well as intrinsic muscle properties (i.e., fiber type composition, calcium kinetics) [14, 15], while maximal strength and muscle size become more influential for late rapid torque measures [14, 16]. Other structural factors influencing RTD include tendon stiffness [17] and pennation angle [12], both of which are negatively affected by age. The majority of studies reporting on age-related changes in rapid torque measures at early and late time intervals examined the knee extensors [11, 18–20]. To the best of our knowledge, only two studies have assessed the plantar flexors (PFs) [6, 12] and the findings for early RTD were equivocal. It is critically important to gain more evidence on the PFs due to the significance of this muscle group for optimal gait in older adults [21], and due to the more mixed fiber type composition of the PFs, age-related changes in neuromuscular function may differ from that of the knee extensors. In addition, RTD during an isotonic contraction of the PFs was recently shown to be a stronger predictor of power output compared to other factors such as velocity, muscle strength or cross-sectional area (CSA) in middle-aged and older males [22]. Considering the importance of muscle power preservation in older adults [23], this lends further support for the need to investigate the effects of aging on rapid torque production, and the factors influencing its decline.

Researchers commonly normalize rapid torque measures by peak torque (PT) (i.e., maximal strength) to indirectly infer the influence of other physiological factors on age-, training-, or fatigue-related changes in rapid torque parameters. Normalized rapid torque measures are believed to be more influenced by qualitative factors such as muscle fiber type composition and neural drive in contrast to maximal strength [3, 14]. However, PT explains up to 78% of the variance in RTD [14], thus these measures share some physiological contributors. Therefore, it is likely more informative to normalize rapid torque measures to muscle size (i.e., CSA). Normalization to CSA enables more accurate dimensional scaling of a physiological determinant of rapid torque production [17], which may be particularly useful if the intent is to more precisely identify contributions from physiological factors. With that said, CSA may only explain ~49% - 59% of the variance in maximal strength of older males [17, 24]. To the best of our knowledge, there are no reports on the age-related differences of rapid torque measures normalized to both CSA and PT, for the PFs. Aside from the influence of CSA and PT, muscle quality via echo-intensity (EI) [25], an ultrasound-derived quantitative gray-scale analysis of fat or fibrous tissue infiltration of muscle, and rate of muscle activation [5, 26] are associated with rapid torque measures as well. Therefore, it is important to assess numerous factors (i.e., muscle CSA and quality, muscle activation) when examining the effects of aging on rapid torque production. The primary purpose of this study was to compare early and late rapid torque parameters of the PFs in middle-aged and older males, as well as to determine the effect of normalization to PT and CSA. In addition, we sought to examine physiological correlates of early and late rapid torque measures. We hypothesized, based on the findings of Gerstner et al. [12], that only late rapid torque measures would be lower in older males, and that normalization to PT and CSA would have no influence on these findings.

## Materials and methods

### Participants

Twenty-nine healthy, middle-aged (n = 14; 45 ± 2 yrs) and older (n = 15; 65 ± 3 yrs) males who reported not having performed structured endurance or resistance training exercise in the past 5 yrs volunteered for this study. All participants were screened via self-reported questionnaire for the following exclusion criteria: presence of unstable cardiovascular, metabolic or renal disease, diagnosed myocardial infarction within the last two-years, terminal illness, a history of cerebrovascular disease, any condition affecting neuromuscular function, an artificial lower-body joint, rheumatoid arthritis, a fracture within the past year, reliance upon an assistive walking device, and Mini-mental State Exam score <23. Participants were recruited from senior centers and the surrounding community through word of mouth, email, and flyer advertisements. This study was approved by the Kennesaw State University Institutional Review Board prior to data collection. All participants provided oral and written consent prior to beginning the study.

### Experimental design

Participants visited the lab on two occasions separated by at least three days but not more than seven. Familiarization with PF testing was completed during the first visit. Ultrasonography and PF testing were performed during the second visit. In addition, participants were instructed to avoid alcohol and vigorous physical activity for 24 and 48 hr, respectively, before each visit.

Torque was recorded during maximal voluntary isometric contractions (MVICs) of the dominant limb using a calibrated Biodex 4 isokinetic dynamometer (Biodex Medical Systems, Inc. Shirley, NY, USA). Leg dominance was determined via inquiry of preferred kicking leg [27]. EMG of the medial gastrocnemius (MG) was recorded using a parallel bar, bipolar surface electrode (Delsys Trigno, Delsys, Inc., Natick, MA, USA). The skin over the muscle was shaved, abraded, and cleaned with alcohol, and subsequently the electrode was placed over the MG muscle belly in accordance with the recommendations of the SENIAM project [28]. Torque as well as surface electromyography (EMG) signals were sampled at 10 kHz using EMGworks software (Delsys, Inc., Natick, MA, USA). Participants were seated with hands across their chest, restraining straps over their trunk, pelvis, and thigh, and the input axis of the dynamometer aligned with the lateral malleolus as the axis of rotation for the ankle. The knee was extended to 170° (full extension = 180°) and hip was maintained at 120°. The foot was secured to the footplate with two straps over the dorsal aspect and a custom ankle wrap technique to anchor the heel to the footplate. Ankle position was set at a neutral angle (neutral = 90°) for MVICs.

### Physical activity

Physical activity (steps per day) was objectively measured via an Actigraph GTX3+ accelerometer (Actigraph, Pensacola, FL, USA). Only data for participants with wear time of at least 10 hrs per day, for a minimum of 3 days was used for analysis, as previously described [22].

### Ultrasonography

Panoramic images of the MG and lateral gastrocnemius were obtained using a B-mode ultrasound (LOGIQ S7, General Electric Company, Milwaukee, WI) using previously reported procedures [22]. Briefly, images were acquired with a multifrequency linear-array probe (ML6-15 L; 5–13 MHz; 50mm field of view; General Electric Company, Milwaukee, WI) using the LogicVIEW function. Three images for each muscle were captured at 1/3 the distance from the tibial articular cleft between the femur and tibia condyle to the lateral malleolus on the dominant leg [29]. The polygon function in ImageJ software (version 1.46r, National Institutes of Health, Bethesda, Md.) was used to select as much of the muscle as possible without including the surrounding fascia. Subsequently, CSA and muscle quality were calculated for each muscle. Muscle quality was determined from EI, assessed via gray-scale analysis using the histogram function. The mean EI was expressed as a value between 0 (black) and 255 (white). Subcutaneous fat thickness for both muscles was measured using the straight line function, and was used to calculate normalized EI as suggested by Young et al. [30]. For statistical analysis, CSA was calculated as the sum of the gastrocnemii muscles, while EI was calculated as the average of the gastrocnemii. The CSA and EI data as well as reliability statistics have been reported elsewhere [22].

### Maximal voluntary isometric contractions (MVICs)

Prior to MVIC testing, participants began by performing two submaximal isometric contractions at 50% and 75% of perceived maximal effort. Participants then performed 3, 5 sec MVICs separated by 2 min of rest. Participants were instructed to push on the footplate as “hard and fast as possible” using only the PFs, while avoiding involvement of the quadriceps. Strong verbal encouragement and visual biofeedback was provided during testing. Participants were instructed to avoid pretension or a countermovement prior to each trial. If pretension or a countermovement was visualized, an additional MVIC was performed. In addition, during subsequent analysis, the slope of torque signal prior (100 ms) to torque onset was determined for each contraction and any MVIC with a slope value that exceeded ± 5 Nm·s^−1^ was discarded [12].

### Signal processing

**Fig 1:**
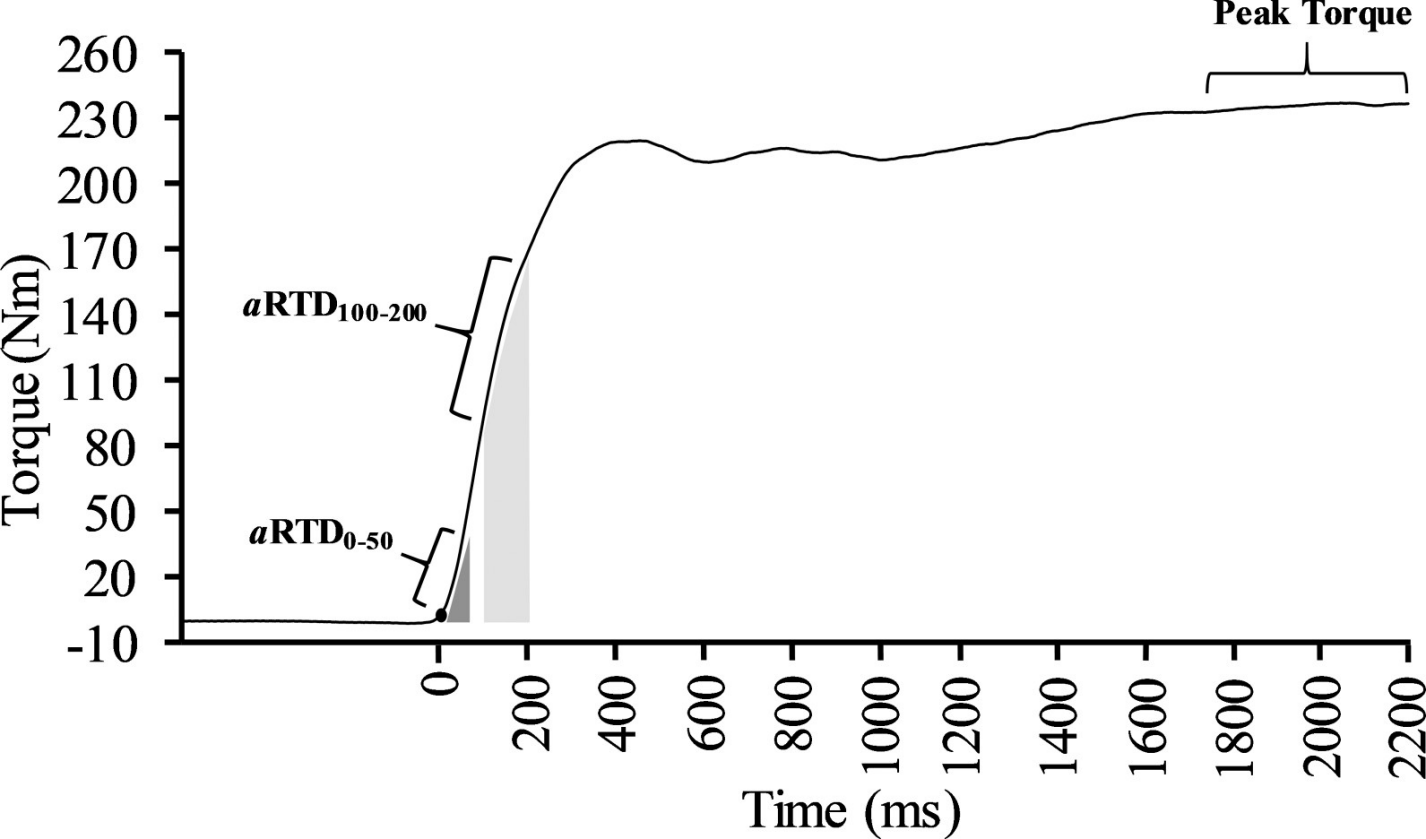
Example of a processed torque signal from a maximal voluntary isometric contraction. Indicated is the portion of the torque-time curve from which rate of torque development from 0 to 50 (*a*RTD_0-50_) and 100 to 200 ms (*a*RTD_100-200_) were calculated as well as impulse from 0 to 50 (dark grey area) and 100 to 200 ms (lighter grey area).

The scaled, gravity corrected torque signal was digitally filtered with a zero lag, low-pass (150 Hz) [31] Butterworth filter using custom written software (LabVIEW, National Instruments, Austin, TX). Peak torque (PT) was considered the highest 500 ms rolling average and was used for normalization of all torque variables. Absolute and normalized torque were recorded at 50 (*a*TQ_50_, *n*TQ_50_), 100 (*a*TQ_100_, *n*TQ_100_), and 200 (*a*TQ_200_, *n*TQ_200_) ms after contraction onset. RTD was derived from the linear slope of the torque-time curve (Δtorque/Δtime) and absolute as well as normalized early RTD was obtained from contraction onset to 50 ms (*a*RTD_0–50_, *n*RTD_0–50_), while late RTD was acquired from 100 to 200 ms (*a*RTD_100–200_, *n*RTD_100–200_) [12]. In addition, each absolute torque measure was made relative to CSA (e.g., “specific” TQ_50_). Impulse was calculated as the area under the torque-time curve (∫Torque d*t*) for the same early (IMP_0-50_) and late (IMP_100-200_) time intervals. Finally, RTD (*a*RTD_0–200_) and impulse (IMP_0-200_) were also calculated from contraction onset to 200 ms to serve as overall measures of rapid torque production. Torque onset was set at 2.5 Nm [19]. The zero means EMG signal was processed using a 4th order Butterworth filter with a low- and high-frequency cutoff of 10 and 500 Hz, respectively. The signal was then smoothed using a zero-lag, low-pass filter (10 Hz) and normalized to its peak amplitude (PEMG). Rate of EMG rise (RER) (i.e., rate of muscle activation) was calculated as the linear slope of the normalized EMG signal at 30 (RER_0-30_), 50 (RER_0-50_), and 75 (RER_0-75_) ms from onset [6, 22]. The EMG onset was determined as the moment when baseline amplitude reached 2.5% of PEMG. The MVIC producing the highest PT was used for subsequent analysis. An example of a processed torque signal from an MVIC can be seen in Fig 1.

### Statistical analyses

Normality of data was assessed within each group using the Shapiro-Wilk Test. Independent samples t-tests were used to make comparisons between groups. However, in the case of a non-normally distributed dependent variable, a Mann-Whitney U test was performed. Levene's test was used to assess homogeneity of variance. Pearson product-moment partial (controlling for age) correlation coefficients were calculated to examine the relationship between ultrasound, RER, and torque variable with groups collapsed. Statistical analyses were performed using PASW software version 26.0 (SPSS Inc., Chicago, IL, USA) and an alpha level of p ≤ 0.05 was used to determine statistical significance. One middle-aged participant was excluded from the physical activity analysis due to not meeting the wear time criteria, and physical activity level data for another middle-aged participant was not obtained due to technical problems. Effect size was reported as Cohen's *d* for independent samples t-tests results and 0.30, 0.50, and 0.80 were used to indicate small, moderate, and large effect sizes, respectively [32]. For non-normally distributed dependent variables, effect size was reported as “*r*” and interpreted using Cohen’s criteria: small effect: ≥ -0.3; moderate effect: < -0.3; large effect: < -0.5 [32]. Correlation coefficients were categorized as weak, moderate, or strong relationships for values of 0.35 or less, 0.36 to 0.67, and 0.68 or more, respectively [33]. All data in text and tables are reported as mean ± SD, while data in figures is displayed as mean ± SEM.

## Results

All dependent variables were normally distributed except RER_0-75_ (p = 0.009) and CSA (p = 0.050) in the middle-aged group, as well as specific RTD_0-50_ (p = 0.043) in the older males. Characteristics for both groups are displayed in Table 1. The groups were similar in height (p = 0.884), body mass (p = 0.448), body mass index (p = 0.335), and physical activity level (p = 0.971). PT was not different between groups (p = 0.105; *d* = 0.63), but *a*RTD_0-200_ (p = 0.024; *d* = 0.89) was lower in the older males (Table 1).

**Table 1:** Characteristics for the middle-aged and older group.

Variable	Middle-aged	Range	Older	Range
Age (yrs)	45.28 (2.64)	41–48	65.33 (3.26)	60–69
Height (cm)	176.97 (8.74)	165–194	177.50 (10.49)	150–192
Body mass (kg)	93.01 (15.67)	69.5–119.7	88.87 (13.24)	64–108.2
BMI (kg‧m^2^)	29.78 (5.24)	23.11–41.67	28.19 (3.34)	22.14–32.53
Steps Per Day	5423.21 (1737.59)	2,941–9,312	5441.62 (2766.86)	2,763–12,271
PT (Nm)	162.56 (37.55)	114.54–236.59	137.06 (43.79)	71.29–214.70
*a*RTD_0-200_ (Nm·s^-1^)	1,259.49 (304.96)	866.60–1,893.14	991.33 (300.92)*	498.49–1,590.49

PT, peak torque; *a*RTD_0-200_, absolute rate of torque development 0–200 ms

* indicates significantly lower in older males

### Cross-sectional area (CSA) and echo-intensity (EI)

CSA (middle-aged: 24.52 ± 6.61 cm^2^ vs. older: 21.59 ± 4.93 cm^2^) and EI (middle-aged: 145.96 ± 10.05 au vs. older: 146.71 ± 12.00 au) were similar between groups (p = 0.295; *r* = -0.19 and p = 0.857; *d* = -0.07, respectively).

### Rapid torque parameters

**Fig 2:**
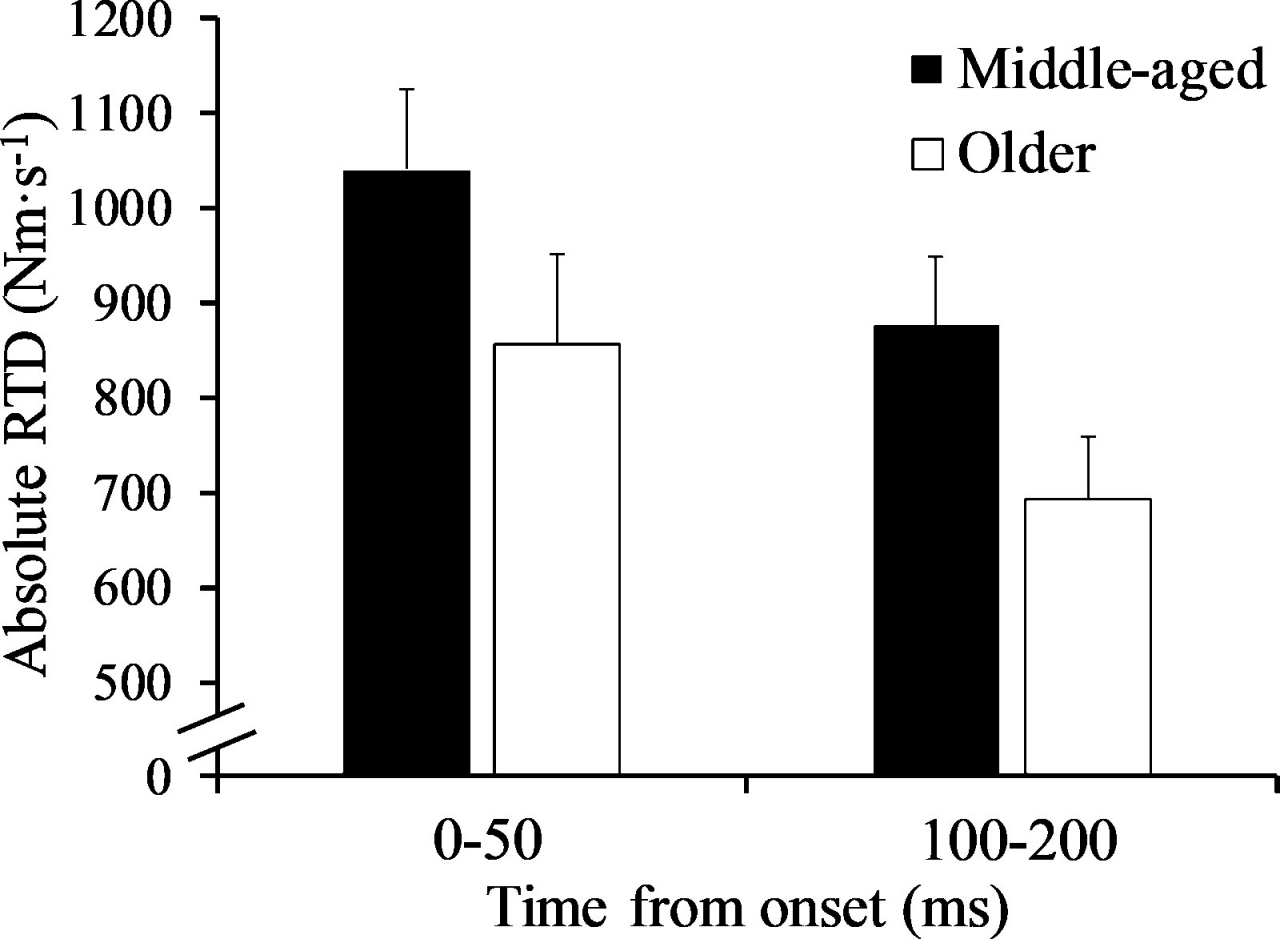
Early (0–50 ms) and late (100–200 ms) absolute rate of torque development (RTD) in the middle-aged and older group.

**Fig 3:**
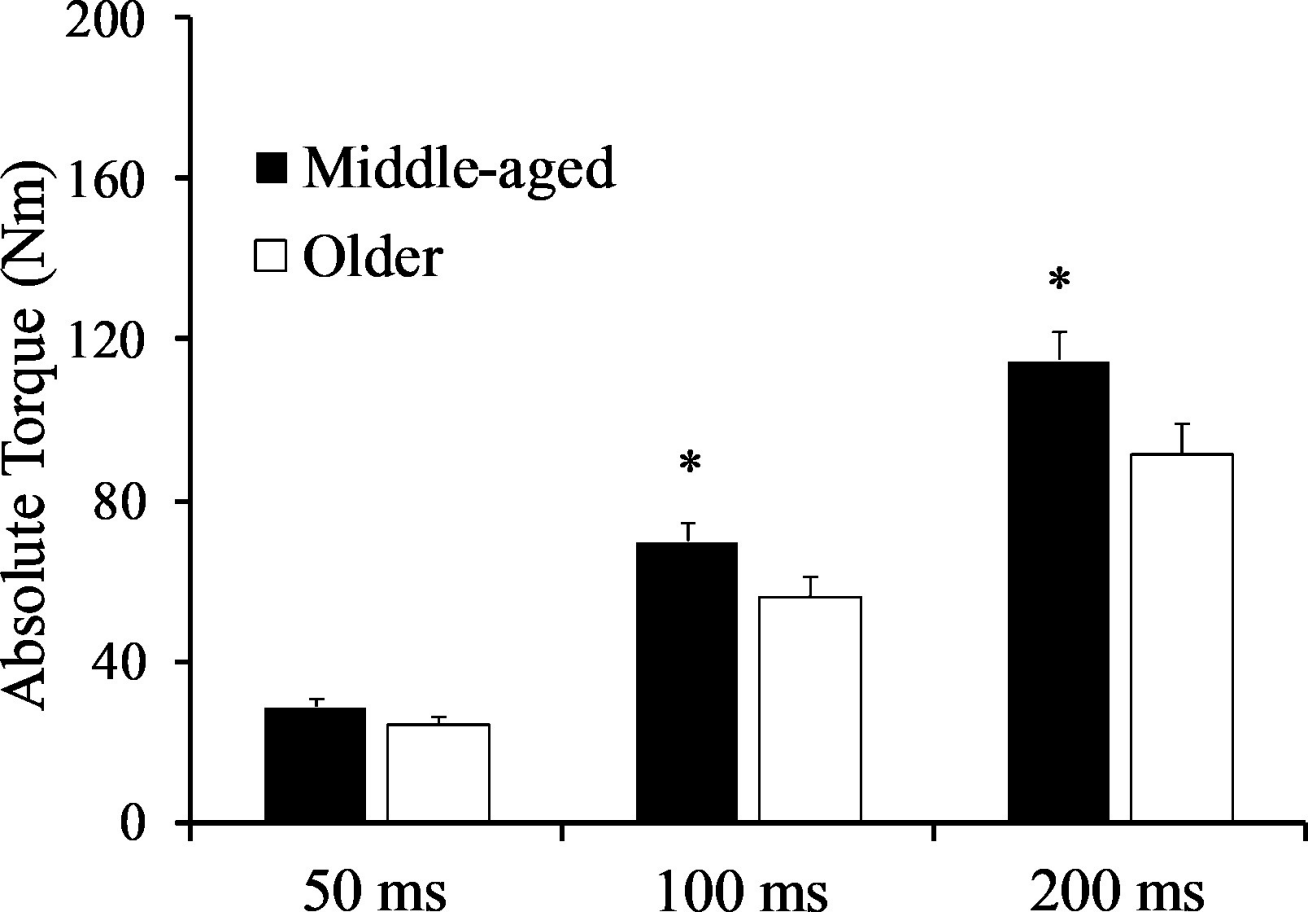
Absolute torque at 50, 100, and 200 ms from onset in both groups. * indicates significantly higher in middle-aged males.

*a*RTD_0-50_ (p = 0.162; *d* = 0.54) and *a*RTD_100-200_ (p = 0.075; *d* = 0.69) were similar between groups (Fig 2). *a*TQ_50_ was similar between groups (p = 0.156; *d* = 0.54), while *a*TQ_100_ and *a*TQ_200_ were lower in the older males (p = 0.046; *d* = 0.77 and p = 0.031; *d* = 0.84, respectively) (Fig 3). IMP_0-50_ was comparable between groups (p = 0.239; *d* = 0.45; middle-aged: 0.32 ± 0.07 vs. older: 0.28 ± 0.09 Nm·s), but IMP_100-200_ (p = 0.032; *d* = 0.84; middle-aged: 4.79 ± 1.11 vs. older: 3.83 ± 1.17 Nm·s) and IMP_0-200_ (p = 0.041; *d* = 0.80; middle-aged: 6.34 ± 1.46 vs. older: 5.12 ± 1.60 Nm·s) were lower in the older males. Torque onset (2.5 Nm), when calculated as a percentage of PT, was not different between groups (p = 0.056; *d* = -0.74; middle-aged: 1.62 ± 0.34 vs. older: 2.03 ± 0.71%PT). There were no differences (p > 0.05) between groups for torque variables normalized to PT (Table 2), nor torque variables normalized to CSA (“specific” variables) (Table 3).

**Table 2:** Torque parameters normalized to peak torque for both groups.

Variable	Middle-aged	Older	Effect Size
*n*TQ_50_	0.18 (0.04)	0.18 (0.07)	-0.13
*n*TQ_100_	0.43 (0.07)	0.43 (0.13)	0.03
*n*TQ_200_	0.71 (0.07)	0.69 (0.15)	0.17
*n*RTD_0-50_	6.45 (1.61)	6.56 (2.53)	-0.05
*n*RTD_100-200_	5.37 (0.96)	5.06 (1.30)	0.27

**Table 3:** Torque parameters normalized to muscle cross-sectional area for both groups.

Variable	Middle-aged	Older	Effect Size
Specific PT	7.00 (2.15)	6.54 (2.35)	0.20
Specific TQ_50_	1.25 (0.52)	1.17 (0.55)	0.15
Specific TQ_100_	3.06 (1.15)	2.72 (1.14)	0.30
Specific TQ_200_	5.00 (1.68)	4.44 (1.78)	0.32
Specific RTD_0-50_	45.93 (20.46)	41.91 (21.70)	0.19
Specific RTD_100-200_	37.67 (12.67)	33.45 (14.96)	0.30

### Rate of EMG rise (RER)

**Fig 4:**
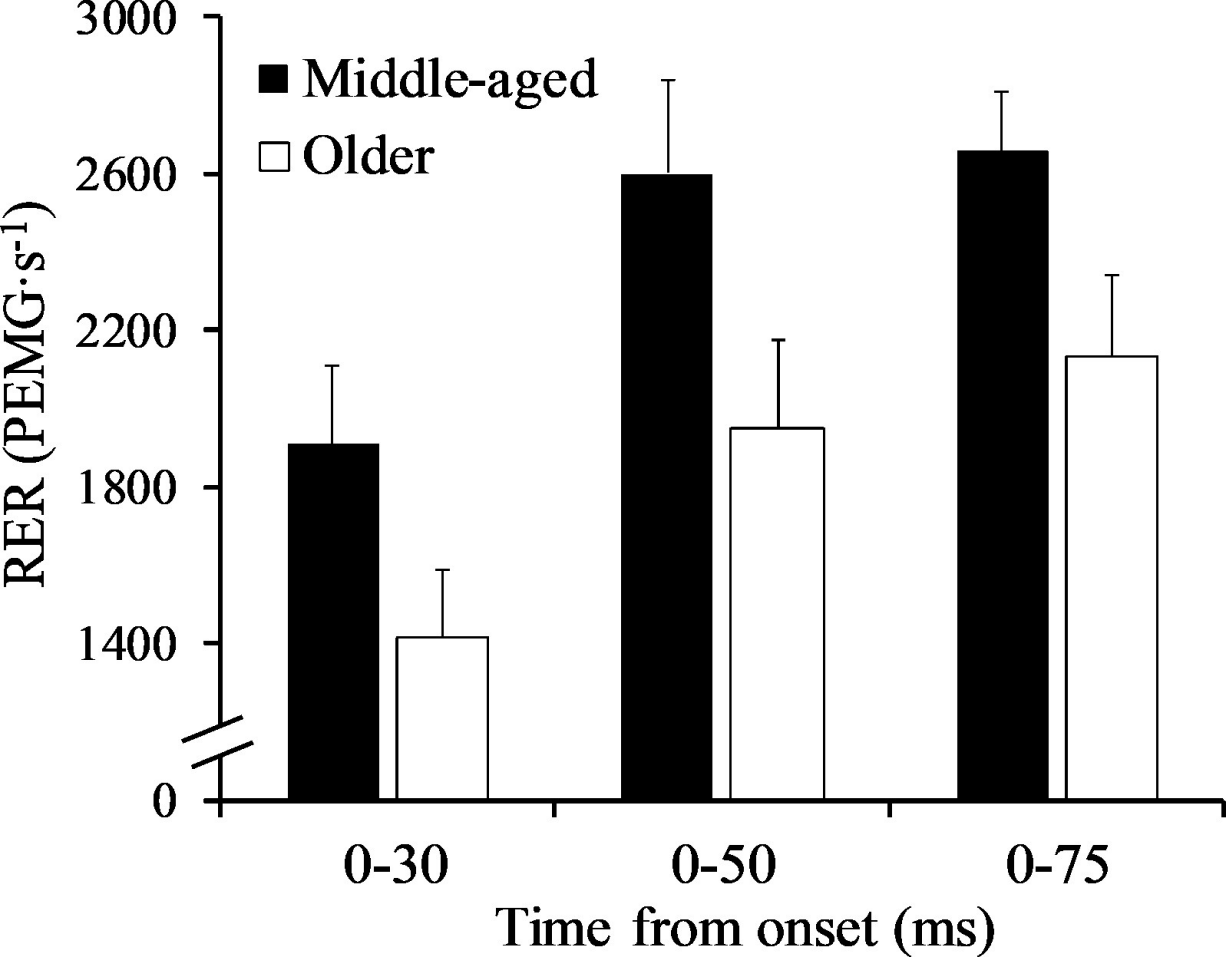
Rate of electromyography rise (RER) at 30, 50, and 75 ms from onset for both groups.

RER_0-30_ (p = 0.072; *d* = 0.69), RER_0-50_ (p = 0.057; *d* = 0.74), and RER_0-75_ ms (p = 0.055; *r* = -0.35) were similar between groups (Fig 4).

### Correlations

All correlation coefficients between rapid torque measures and physiological determinants can be seen in Table 4. CSA was not correlated with PT (*r* = 0.21; p = 0.267) or any early or late rapid torque parameters. EI was moderately correlated with normalized torque parameters only. Only RER_0-75_ was moderately correlated with early absolute torque parameters, while RER at all time intervals was moderately correlated with the majority of rapid torque variables when made relative to PT or CSA.

**Table 4:** Partial correlation coefficients between torque variables and physiological determinants.

		CSA	EI	RER_0-30_	RER_0-50_	RER_0-75_
Absolute	*a*RTD_0-50_	-0.17	-0.31	0.30	0.33	0.43*
	*a*TQ_50_	-0.12	-0.30	0.30	0.33	0.43*
	*a*IMP_0-50_	-0.17	-0.34	0.33	0.36	0.46*
	*a*RTD_100-200_	0.11	-0.00	-0.21	-0.22	-0.17
	*a*TQ_100_	-0.10	-0.22	0.17	0.19	0.30
	*a*TQ_200_	-0.01	-0.14	0.01	0.02	0.11
	*a*IMP_100-200_	-0.17	-0.18	0.06	0.08	0.19
Normalized	*n*RTD_0-50_	-0.31	-0.45*	0.46*	0.52**	0.64**
	*n*RTD_100-200_	-0.08	-0.06	-0.02	-0.07	-0.08
	*n*TQ_50_	-0.32	-0.44*	0.47*	0.52**	0.64**
	*n*TQ_100_	-0.32	-0.38*	0.39*	0.44*	0.56**
	*n*TQ_200_	-0.32	-0.38*	0.35	0.39*	0.52**
Specific	Specific PT	-0.55**	0.01	0.20	0.21	0.19
	Specific RTD_0-50_	-0.60**	-0.25	0.47*	0.50**	0.56**
	Specific RTD_100-200_	-0.55**	-0.06	0.18	0.17	0.17
	Specific TQ_50_	-0.64**	-0.25	0.48**	0.51**	0.57**
	Specific TQ_100_	-0.66**	-0.19	0.43*	0.46*	0.51**
	Specific TQ_200_	-0.65**	-0.15	0.36*	0.38*	0.41*

*Correlation is significant at the 0.01 level

**Correlation is significant at the 0.05 level

## Discussion

The purpose of this study was to compare early and late rapid torque parameters of the PFs in middle-aged and older males, as well as to determine the effect of normalization to PT and CSA. In addition, we sought to examine physiological correlates of early and late rapid torque measures. Similar to previous research, rapid torque characteristics were more dramatically affected by aging compared to PT [5–7]. The modest age group difference (~20 yrs) in this study and finding that maximal strength was similar between groups further endorses this notion. In particular, late rapid torque parameters were lower in older males, whereas early rapid torque measures were similar between middle-aged and older males. Group differences were eliminated when rapid torque parameters were made relative to both PT and CSA, which may indicate that maximal strength and muscle size had an influential role for the age-related differences in absolute, late rapid torque measures.

Our findings indicated that late rapid torque parameters were more influenced by age compared to earlier rapid torque characteristics of the PFs. More specifically, while *a*RTD_0-50_ and *a*TQ_50_ were similar between groups, *a*TQ_100_, *a*TQ_200_, as well as late impulse (i.e., IMP_100-200_) were lower in older males. Interestingly, in contrast to IMP_100-200_, *a*RTD_100-200_ was not different between groups even with the moderate effect size found (*d* = 0.69). It should also be noted that “overall” rapid torque production (i.e., 0–200 ms) was lower in older males, which was likely due to the age-related diminishment in quick torque production 100 ms after contraction onset and later. Our findings are similar to those of Gerstner et al. [12], in which late but not early rapid torque measures were negatively influenced by age, although young and older males were examined in that study. The previous study that compared RTD of the PFs at different time intervals in middle-aged and older adults determined that both, early and late absolute RTD were lower in the older group [6]. The age-related decrease in the current study for *a*RTD_100-200_ is similar to that reported by Thompson et al. [6] (20.3% vs. 24.3%). The discrepancy for the early RTD (i.e., *a*RTD_0-50_) findings may be due to differences in physical activity levels of participants. The present findings should primarily represent the effects of aging due to the absence of self-reported structured exercise and similar physical activity levels between groups. Another possible explanation for the discrepancy related to the early RTD findings could be the difference in the automated torque onset used for determining RTD, as a 4 Nm onset was used by Thompson et al. [6] while a 2.5 Nm onset was used in the present study. Rapid torque measures will vary depending on whether an automated or manual torque onset is used since the onset tends to occur earlier in the torque signal for a manual onset [12, 34]. For example, Gerstner et al. [12] demonstrated that, although there was no difference when a manual onset was used, *a*TQ_50_ was significantly different between young and older males when an automated 4 Nm onset was used. Regardless of the onset value, the use of an automated onset is limited in its ability to capture initial torque production (i.e., 20–30 ms) [34], although it is important to note that the torque onset used in the present study, when calculated as a percentage of PT, was not different between groups. Nevertheless, few studies have specifically examined age-related changes in early and late rapid torque parameters [6, 12, 18, 19], and while some findings are in disagreement [6], other reports in conjunction with the present findings suggest that rapid torque production during the late phase of muscle contraction, in particular, may be most dramatically affected by aging [12, 18, 19]. Considering the unique physiological factors influencing early and late rapid torque production [14, 16], this hypothesis warrants further investigation as it could prove insightful for identifying optimal interventions.

CSA of the PFs is smaller in older adults compared to their younger counterparts [35, 36], but contrasting findings have been reported [12]. The moderate age difference between groups in the current study is likely the reason CSA and PT were similar between groups, but the moderate effect size for PT (*d* = 0.63) is notable. Indeed, while CSA was not correlated with PT or any of the rapid torque measures, PT was strongly associated with all absolute late rapid torque parameters (*r* = 0.68–0.82). For the current study, we suspected that any potential age-related differences in PT or CSA would be too modest to be influential for rapid torque production. However, there were no longer age-related differences for any torque variables when made relative to PT or CSA. These findings support the notion that CSA and PT had a mediating role for the age-related difference in late rapid torque production. RTD normalized to PT has either been similar [37] or lower [12] (late RTD only) in older adults for the PFs. In contrast with our findings, Thompson et al. [6] reported lower normalized early and late RTD in older males compared to middle-aged males. While variation in the calculation of RTD may partially explain this discrepancy, it is noteworthy that in contrast to the current study, the older males demonstrated a lower PT (-27.6%) compared to their middle-aged counterparts in Thompson et al. [6]. The relatively homogenous groups used in this study was also likely influential for the discrepancy in normalized RTD findings between studies. Our finding that PT was similar between groups is not necessarily surprising since previous research has demonstrated little to no change in PT of the PFs from the 5^th^ to 7^th^ decade [38, 39]. Nonetheless, the similarities in normalized and specific (i.e., relative to CSA) rapid torque parameters suggest that qualitative properties such as tendon stiffness [17], EI [12], and muscle fiber type composition [15] were not substantially different between groups.

To the best of our knowledge, only one study has examined the association among early and late rapid torque parameters with RER and EI of the PFs [12]. It was determined that muscle activation during the first 200 ms of contraction was correlated with RTD during the corresponding time intervals [12]. Similarly, positive correlations were found for RER_0-75_ and early rapid torque production in the present study, which is in-line with previous work demonstrating that initial muscle activation primarily influences early torque production [16, 40]. Gerstner et al. [12] reported lower muscle activation for the PFs in older compared to young males during the late phase of muscle contraction (100–200 ms), in particular, although differences in the age groups and procedures used to calculate muscle activation make comparisons to Gerstner et al. [12] difficult. Unlike the current study, Thompson et al. [6] found RER of the PFs to be lower in older males compared to their middle-aged counterparts. The greater difference in age between groups or larger sample size may be responsible for the discrepancy between studies. While muscle architecture was not assessed in the present study, it is possible that age-related differences in pennation angle may explain the diminished late-phase, absolute rapid torque production in older males [12]. In regards to EI, Gerstner et al. [12] reported higher EI (i.e., decreased muscle quality) for the MG in older compared to young males, but our findings demonstrate very similar values between middle-aged and older males. In addition, EI was inversely associated with only normalized rapid torque measures, which is not surprising since these parameters are believed to be indicative of qualitative muscle factors [12, 14]. The age difference between groups in the current study was likely too modest for EI to be influenced, especially if infiltration of non-contractile adipose or connective primarily results from myofiber apoptosis [41] since this should not be prevalent in the 7^th^ decade. Previously, EI of the quadriceps [42] and MG [12] was found to be correlated with absolute RTD, however, Gerstner et al. [12] reported associations with late absolute RTD only. Although only correlational, findings from previous work may indicate that the age-related increase in non-contractile adipose or connective tissue [43] inhibits muscle fiber shortening [44] and subsequently rapid torque production [45].

The current study had a few limitations. While the comprehensiveness of this study is a feature, it should be noted that the cross-sectional design is more susceptible to confounding variables compared to a longitudinal design. However, the objective measurement of physical activity level was a strength of this study, which addressed a common confounding factor for age-related studies. Although the soleus appears to be well preserved with aging relative to the gastrocnemii muscles [46], it is important to note that we did not include the soleus in our calculation of PF CSA. In addition, interpretation is limited to males only, especially since the age-related decline in some neuromuscular properties can differ between sexes. Future research investigating rapid neuromuscular function should make considerations for muscle-specific differences, age of the study sample, as well as the procedures for calculating RTD. For example, the use of brief (~1 sec) rapid isometric contractions has been recommended for the study of RTD [34], but the present study derived RTD from a longer duration MVIC which could have underestimated RTD. Finally, some caution is needed for interpretation of these findings due to our relatively small sample size.

Our findings provide additional support for previous work showing that rapid torque measures are more dramatically influenced by aging compared to maximal strength [5–7]. In addition, our results demonstrated that rapid torque production was preferentially impaired during the late phase of muscle contraction in older males compared to their middle-aged counterparts. However, differences between groups disappeared when torque parameters were normalized to peak torque and gastrocnemii cross-sectional area, suggesting the amount of contractile tissue and maximal force generating capacity was likely the primary reason for the age-related difference in late rapid torque production. The lack of association between late rapid torque measures with qualitative properties (i.e., echo-intensity, rate of muscle activation) provided further support for this conclusion.
